# Interfacial Concentrations of Hydroxytyrosol Derivatives in Fish Oil-in-Water Emulsions and Nanoemulsions and Its Influence on Their Lipid Oxidation: Droplet Size Effects

**DOI:** 10.3390/foods9121897

**Published:** 2020-12-18

**Authors:** Marlene Costa, Sonia Losada-Barreiro, Carlos Bravo-Díaz, Luís S. Monteiro, Fátima Paiva-Martins

**Affiliations:** 1 REQUIMTE-LAQV, Department of Chemistry and Biochemistry, Faculty of Sciences, University of Porto, 4169-007 Porto, Portugal; marlene.andreia.costa@gmail.com (M.C.); sonia@uvigo.es (S.L.-B.); 2 Departamento de Química-Física, Facultade de Química, Universidade de Vigo, 36310 Vigo, Spain; cbravo@uvigo.es; 3 Chemistry Centre, University of Minho, Gualtar, 4710-057 Braga, Portugal; monteiro@quimica.uminho.pt

**Keywords:** droplet size, antioxidant efficiency, interfacial concentration, hydroxytyrosol, emulsions, nanoemulsions, pseudophase kinetic model

## Abstract

Reports on the effect of droplet size on the oxidative stability of emulsions and nanoemulsions are scarce in the literature and frequently contradictory. Here, we have employed a set of hydroxytyrosol (HT) esters of different hydrophobicity and fish oil-in-water emulsified systems containing droplets of different sizes to evaluate the effect of the droplet size, surfactant, (Φ_I_) and oil (Φ_O_) volume fractions on their oxidative stability. To quantitatively unravel the observed findings, we employed a well-established pseudophase kinetic model to determine the distribution and interfacial concentrations of the antioxidants (AOs) in the intact emulsions and nanoemulsions. Results show that there is a direct correlation between antioxidant efficiency and the concentration of the AOs in the interfacial region, which is much higher (20–200 fold) than the stoichiometric one. In both emulsified systems, the highest interfacial concentration and the highest antioxidant efficiency was found for hydroxytyrosol octanoate. Results clearly show that the principal parameter controlling the partitioning of antioxidants is the surfactant volume fraction, Φ_I_, followed by the O/W ratio; meanwhile, the droplet size has no influence on their interfacial concentrations and, therefore, on their antioxidant efficiency. Moreover, no correlation was seen between droplet size and oxidative stability of both emulsions and nanoemulsions.

## 1. Introduction

Interest in using fish oils to prepare foods comes from their high content of long chain omega-3 fatty acids present in their triacylglycerols, especially docosahexaenoic acid (DHA) and eicosapentaenoic acid (EPA) [1]. These omega-3 polyunsaturated fatty acids (PUFAs) have shown clear health benefits to consumers and therefore, incorporation of fish oil into foods has been increasing particularly in countries where there is a shortage of fish [1]. However, their incorporation decreases the oxidative stability of foods since EPA and DHA are quickly oxidized. This is due to the high number of bis-allylic hydrogens bonded in their chemical structure with high susceptibility to homolytic breakdown and consequent formation of radicals [2].

This oxidation reaction, leading to the rancidity of the oil and to the production of harmful products, represents, after microbial spoilage, the major cause of food deterioration and rejection by both consumers and industry [3,4]. Delaying the oxidative reactions of PUFAs has become a major task and constitutes one of the most studied areas in food science and technology. Oxidation has a direct implication on the sensory, nutritional quality, safety, lifetime and on the commercial value of food products [5,6] since the reactions involved are related to the production of both toxic compounds and substances that impart undesirable aromas and colors to foods [7].

**Figure 1:**
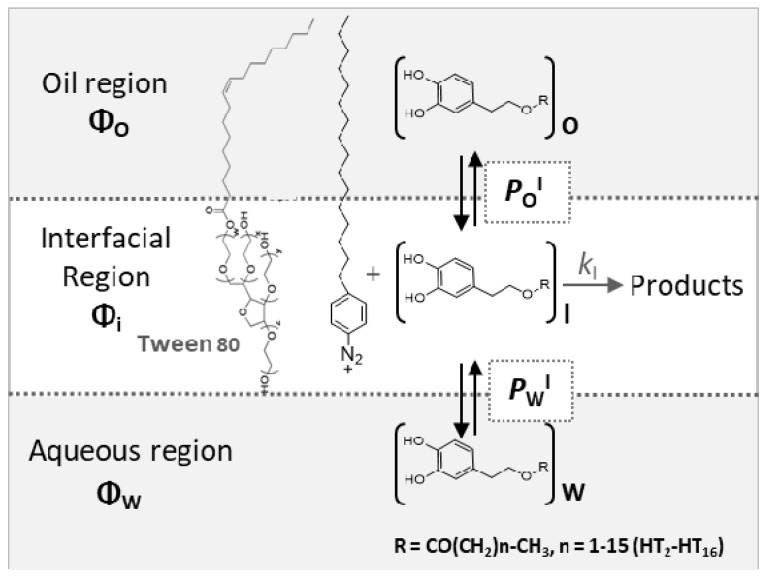
Partition of hydroxytyrosol (HT) and its ester derivatives (AO) between the different regions of a model emulsified system. *P*_O_^I^ = partition constant of the AO between oily region and interfacial region, *P*_W_^I^ = partition constant of the AO between aqueous region and interfacial region, and *k*_I_ = rate constant for the reaction between 16-ArN_2_^+^ and the AO in the interfacial region. Φ refers to the volume fraction of an aqueous (Φ_W_), interfacial (Φ_I_), and oil (Φ_O_) regions.

Currently, there are several methods capable of retarding lipid oxidation: preventing access to oxygen, lowering the temperature, or correct packaging. The most effective and cheapest method is the use of antioxidants (AOs) [8,9,10,11]. Efficient antioxidants are those molecules which trap lipid radicals faster than they are produced, i.e., *r*_inh_ = *k*_inh_ [LOO^•^][AO] >> *r*_p_ = *k*_p_[LOO^•^][LH] (LOO^•^ stands for a peroxyl radical, LH is a lipid, *k*_inh_ and *k*_p_ are the inhibition and propagation rate constants, respectively) [12,13]. Nevertheless, choosing an efficient AO is challenging because its efficiency in emulsions depends on both the rate constant for the scavenging reaction, *k*_inh_, and its effective concentration in the interfacial region [12,14] (Figure 1).

By applying the pseudophase kinetic model, we previously determined the effective concentration of AOs in the interfacial region of intact emulsified systems, thus demonstrating that there is a direct relationship between the interfacial concentrations and their antioxidant efficiencies [12,14,15]. The pseudophase kinetic model assumes that antioxidants partition thermodynamically between the different regions of emulsions and are in dynamic equilibrium [15]. Thus, their effective concentration in each region depends on the solvent properties of each region but not on the size of droplets. The advantages, limitations, assumptions, and mathematical treatment of the model are discussed in detail elsewhere [16,17].

Here, we have employed the formalism of the pseudophase kinetic model to investigate the effects of droplet sizes on the distribution of antioxidants derived from hydroxytyrosol (HT, Figure 1) in oil-in-water nanoemulsions and thus, on their antioxidant efficiency. Commercial emulsions can present a wide variety of droplet sizes, from the nanometer-sized emulsions prepared for parenteral nutrition (~150 nm), to macroemulsions commonly found in dressings (>1000 nm). However, literature reports regarding the effects of the droplet size and interfacial area on their oxidative stability are scarce and sometimes contradictory. Some researchers found no effects of the droplet size on lipid oxidation rate [5,18,19], meanwhile others have concluded that “emulsions with smaller droplets are more easily oxidized” [20,21,22,23,24]. Thus, more research is necessary to assess the role of droplet size on the oxidative stability of emulsions.

Based on our previous results and on the application of the pseudophase kinetic model, which presumes that droplet size does not affect antioxidant distributions and hence antioxidant efficiencies, [15,25,26,27] we hypothesize that the interfacial antioxidant concentrations are not affected by the droplet size. To prove, or discard, our hypothesis, we undertook a thorough investigation by employing fish oil-in-water emulsions and nanoemulsions with the same chemical composition but different droplet sizes (ranging from ~166 nm to ~4600 nm). In the intact emulsions and nanoemulsions, the effective concentrations of a series of hydroxytyrosol derivatives bearing the same reactive moiety but of different hydrophobicity was determined (Figure 1). The main aim of this work is to get a better understanding on how the droplet size affects the antioxidant distribution in the oil, interfacial and aqueous regions of emulsified systems and how relevant parameters control the antioxidant efficiency.

## 2. Materials and Methods

### 2.1. Materials

Tween 80, citric acid, and *N*-(1-naphthyl) ethylenediamine (NED) were purchased from Acros Organics (Fair Lawn, NJ, USA). Mili-Q grade water was employed in all experiments. Commercial fish oil (generously provided by Biomega Natural Nutrients S.L., Boiro, Spain) was stripped from endogenous antioxidants by passing it twice through a Al_2_O_3_ column under nitrogen [28]. The fatty acid composition of the fish oil, expressed as % total fatty acids, was 52% PUFAs (among others 16% EPA, 29% DHA), 25% MUFA (monounsaturated fatty acids), and 23% SFA (saturated fatty acids). Hydroxytyrosol was purchased from Seprox Biotech (Murcia, Spain). HT esters (Figure 1; acetate, HT2; hexanoate, HT6, octanoate, HT8; dodecanoate HT12; and hexadecanoate HT16) were prepared according to Almeida et al. [27]. 4-Hexadecylbenzenediazonium tetrafluoroborate, 16-ArN_2_BF_4_, was prepared from 4-hexadecylaniline (Aldrich, 97%) [17].

### 2.2. Preparation of Emulsions and Nanoemulsions

Coarse 1:9 O/W and 4:6 O/W emulsions (10 mL) stabilized with Tween 80 were prepared by mixing the appropriate amounts of stripped fish oil, aqueous acid buffered solution (0.04 M citrate buffer, pH 3.65), and surfactant Tween 80. The volume fraction of surfactant, Φ_I_, defined hereafter as Φ_I_ = V_surf_/V_emulsion_ was varied from Φ_I_ = 0.005 to Φ_I_ = 0.04. The same amount of AO per amount of oil to be protected in all emulsified systems was used in the evaluation of the antioxidant efficiency, i.e., [AO_T_] = 0.125 mM or 0.500 mM in 1:9 W/O and in 4:6 W/O emulsified systems, respectively. In the determination of antioxidant distribution, [AO_T_] = 2 mM. The resulting O/W mixtures were stirred with a Polytron PT-1600 homogenizer (20.000 rpm, 1 min; HEn, high energy method) to produce 1:9 and 4:6 O/W coarse emulsions. Each coarse emulsion (1:9 and 4:6, O/W) was vortexed every 12 h for 1 min to reduce any potential effect of phase separation on the oxidation kinetic profile. No significant phase separation was observed.

1:9 OW emulsions with a Φ_I_ = 0.005 were also prepared with the aid of a vortex (LEn, low energy method).

1:9 O/W nanoemulsions were prepared passing the prepared HEn coarse 1:9 O/W emulsions through a high-pressure homogenizer (Nozzle Z5, Nano DeBEE, Bee International USA, San Diego, CA, USA) at 25,000 psi for 3 cycles. To the naked eye, no creaming or phase separation was observed during storage [25].

### 2.3. Average Droplet Size, Polydispersity, and ζ-Potential Measurements of the Prepared Emulsions and Nanoemulsions

As in previous works, the droplet size, ζ-potential (the electrical potential at the slipping interface), and polydispersity (as measured by the polydispersity index, defined as the mass average over the number average particle radius) of fresh HEn 4:6 O/W emulsions and 1:9 O/W emulsions and nanoemulsions were determined by employing a dynamic light scattering (DLS) Zetasizer (NanoZS laser diffractometer Malvern Instruments Ltd., Worcestershire, UK) at T = 25 °C [15].

As droplet sizes over 5000 nm cannot be measured by DLS, droplet size of LEn 1:9 (O/W, Φ_I_ = 0.005) emulsions loaded with antioxidants were evaluated by employing a turbidimetric method as described by Pearce and Kinsella [29]. These emulsions were obtained by vortexing the components with a vortex apparatus (Fisherbrand™ Analog Vortex Mixer, Thermo Fisher Scientific, Suwanee, GA, USA) at the speed of 3200 rpm during 1 min. The emulsions were then diluted to 1:1000 (*v/v*) and the absorbance of freshly diluted emulsions was measured spectrophotometrically at λ = 500 nm. The absorbance values were used to determine the interfacial area (IA) of the dispersed oil phase according to Cameron [30] and expressed as m^2^/mL of emulsion. The calculated interfacial areas (IA) (Figure S1, Supplementary Data) were then used to calculate the average droplet size of these emulsions [30].

### 2.4. Cyclic Voltammetry

A Hi-Tek potentiostat, type DT 2101, and a Hi-Tek wave generator type PPRl, connected to a Philips recorder, type PM 8043, and to a three electrode, home-built glass cell were used. The working electrode was a vitreous carbon disc with a diameter of 3 mm, the counter electrode was a platinum spiral and the reference electrode was a mercury pool separated from the working electrode by a Luggin capillary.

Stock solutions of HT and its derivatives (10 mM) were prepared by dissolving the appropriate amount in ethanol. The voltammetric working solutions were prepared, in the electrochemical cell, by diluting 0.1 mL of the stock solution in 10 mL of supporting electrolyte in order to obtain a final concentration of 0.1 mM. The pH 3.65 supporting electrolyte was prepared by adjusting the pH of 0.1 M potassium dihydrogen phosphate with phosphoric acid. Emulsions were prepared by diluting 0.1 mL of the stock solution in 10 mL of supporting electrolyte and adding 0.1 mL of a 0.08 g/mL solution of tween 80. The solution was placed in an ultra-sonic bath for 1 min and then added to the electrochemical cell.

The experimental conditions were: scan rate (V/s) = 0.1; starting potential (V) = −0.2; first vertex potential (V) = 0.8; second vertex potential (V) = −0.2. At the end of each experiment, the potential of the mercury pool used as reference electrode was measured versus a Ag–AgCl electrode. Between experiments, the working electrode was repolished with alumina powder (0.05 ~μm).

### 2.5. Efficiency of HT and Its Derivatives in Fish Oil Emulsions and Nanoemulsions

The antioxidant efficiency was determined, as in previous works, [12,15] by monitoring the spontaneous formation of primary oxidation products (conjugated dienes, CDs) with time in the absence (control) and in the presence of AOs. Nanoemulsions were placed in an oven and allowed to spontaneously oxidize at T = 40 °C in the dark. At selected times, 25 µL of each nanoemulsion or emulsion were diluted with ethanol and the absorbance measured at λ = 233 nm. All runs were performed in triplicate. Efficiency is given as the time required to achieve an increase of 0.5% in the conjugated diene content, (Δ%CD = 0.5%). The longer the time, the more efficient the antioxidant. In order to evaluate the antioxidant efficiency of compounds in different emulsified systems at the same time, the relative increase in the oxidative stability was used (Equation (1)):

Relative increase in the oxidative stability = (*t*_(AO)_ ‒ *t*_(C)_)/*t*_(C)_, (1)

being *t*_(AO)_ and *t*_(C)_ the time necessary for the samples loaded with antioxidant and without AO (control) to increase in 0.5% the formation of conjugated dienes, respectively.

### 2.6. Determining Antioxidant Distribution and Local Concentrations in Intact Emulsified Systems

As described in previous works, the distribution of antioxidants in the different regions of the emulsified systems was determined by employing a chemical kinetic method [17]. This is based on the reduction of the probe 16-ArN_2_^+^ by the AO at different Φ_I_, whose reactive group (-N_2_^+^) is located exclusively in the interfacial region (Figure 1 and Scheme S1, Supplementary Data). The experimental (*k*_obs_, Φ_I_) pairs of data were analyzed according to the pseudophase kinetic model [17]. Equations (2) and (3) define the thermodynamic partition constants of the AO between the oil-interfacial, *P*_O_^I^ and aqueous-interfacial, *P*_W_^I^, regions and Equation (4) shows the variation of the observed rate constant, *k*_obs_, for the reaction between the chemical probe and AOs with Φ_I_, from where *P*_W_^I^ and *P*_O_^I^ can be determined:

(2) $${P_{O}}^{I}=\frac{{(\mathrm{AO}}_{I})}{{(\mathrm{AO}}_{O})},$$

(3) $${P_{W}}^{I}=\frac{{(\mathrm{AO}}_{I})}{{(\mathrm{AO}}_{W})},$$

(4) $$k_{obs}=\frac{[{\mathrm{AO}}_{T}]k_{I}{P_{W}}^{I}{P_{O}}^{I}}{\Phi_{O}{P_{W}}^{I}+\Phi_{{}_{I}}{P_{W}}^{I}{P_{O}}^{I}+\Phi_{W}{P_{O}}^{I}}.$$

In Equations (2) and (3), (AO_I_), (AO_O_), and (AO_W_) refer to the effective antioxidant concentration in the interfacial, oil, and aqueous regions (moles per liter of the particular region, mol L^−1^), respectively. In Equation (4), [AO_T_] stands for the stoichiometric AO concentration in the emulsion (moles per liter of emulsion, mol L^−1^), *k*_I_ is the rate constant for the reaction between 16-ArN_2_^+^ and the AO in the interfacial region and Φ_O_, Φ_I_, Φ_W_, are the volume fractions of oil, surfactant and water, respectively. The values of *P*_W_^I^ and *P*_O_^I^ are determined by combining a single set of kinetic experiments (fits of *k*_obs_ vs. Φ_I_) with the value for the partition constant between oil and water in the absence of emulsifier (*P*_W_^O^ = *P*_W_^I^/*P*_O_^I^), and solving two equations for two unknowns. Details on the determination of the partition constants *P*_W_^O^ in binary systems, of the observed rate constants, *k*_obs_ (Figures S2 and S3, Supplementary Data), and simplifications employed for determining the partition constants for water-insoluble and oil-insoluble AOs in emulsified systems can be found elsewhere [16,17].

Once the *P*_W_^I^ and *P*_O_^I^ values are known, the percentage of AO (%AO_I_) and its effective interfacial concentration (AO_I_) can be determined by using Equations (5) and (6). The equations to determine the antioxidant percentage and the concentrations in the oil and aqueous regions of the emulsified systems are not indicated, but can be found elsewhere [17,20]:

(5) $${\%\mathrm{AO}}_{I}=\frac{100\Phi_{I}{P_{W}}^{I}{P_{O}}^{I}}{\Phi_{O}{P_{W}}^{I}+\Phi_{{}_{I}}{P_{W}}^{I}{P_{O}}^{I}+\Phi_{W}{P_{O}}^{I}},$$

(6) $${(\mathrm{AO}}_{I})=\frac{[{\mathrm{AO}}_{T}]\left({\%\mathrm{AO}}_{I}\right)}{\Phi_{I}}.$$

### 2.7. Statistical Analysis

The kinetic experiments were run in triplicate for 2–3 t_1/2_ and *k*_obs_ values varied within ±7–9%, with *r* > 0.995. The anodic potential of each compound, the CD content, droplet size, and polydispersity of the prepared emulsified systems were determined, at least, in triplicate. SPSS 21.0 software was employed for statistical analysis by ANOVA. The significant differences among means were analyzed with Duncan’s multiple range test on a 95% confidence level (*p* ≤ 0.05). Data are displayed as mean ± standard deviation.

## 3. Results and Discussion

### 3.1. Droplet Size and Polydispersity of the Prepared Emulsified Systems

As expected, LEn 1:9 O/W emulsions prepared with Φ_I_ = 0.005 showed droplet sizes above 5000 nm (Table 1). Hydroxytyrosol did not significantly affect the mean droplet size values when compared with the control. However, the more lipophilic esters caused a general increase in the interfacial area values in emulsions loaded with HT derivatives in comparison with the reference (*p* < 0.05) and, therefore, a decrease in the mean droplet size (Table 1). Since emulsions containing HT esters prepared with the aid of a vortex have a higher interfacial area and, therefore, smaller droplets than the control, one can conclude that compounds must take part of the interfacial film, favoring the formation of the oil droplet interface. In fact, there is a decrease in the mean droplet size from the acetate to the octanoate derivative and then an increase up to the hexadecanoate derivative. The mean droplet diameter calculated for these emulsions (Φ_I_ = 0.005) prepared by the aid of the vortex apparatus was ~8500 nm for control and HT loaded emulsions and ~5000 nm for the HT8 loaded emulsions, a range of diameters usually found in many food emulsions.

The HEn 1:9 O/W emulsions (obtained using the polytron homogenizer at 20.000 rpm, 1 min) were quite polydisperse (PDI index = 0.4–0.6), with average droplet sizes of ~1200 and ~478 nm at Φ_I_ = 0.005 and Φ_I_ = 0.02, respectively. Moreover, for Φ_I_ = 0.005, emulsions loaded with HT derivatives did not show significant variation in the droplet size (Table 1). Besides the observed trend towards a lower droplet diameter value for the octanoate loaded emulsion, in emulsions prepared with the aid of a Polytron homogenizer, we could not observe significant difference in the I.A. (*p* < 0.05) between emulsions loaded with different AOs (data not shown). The non-measurable difference in the surface area determined by the turbidimetric method is due to the small variations found in the droplet diameter in the emulsions containing AO prepared with the polytron homogenizer (in the range 1100–1200 nm, determined by DLS).

In the case of coarse 4:6 O/W emulsions, also obtained using the polytron homogenizer, they were also quite polydisperse (PDI index = 0.4–0.8), with average droplet sizes of ~4700 and ~2700 nm at Φ_I_ = 0.005 and Φ_I_ = 0.02, respectively (Table 2). During storage, there was no phase separation until samples were heavily oxidized.

The 1:9 O/W nanoemulsions showed small polydispersity (PDI <0.2), with a monomodal droplet size distribution with average droplet sizes of d ~304 nm (Φ_I_ = 0.005), ~231 nm (Φ_I_ = 0.01) and ~166 nm (Φ_I_ = 0.02) (Table 2). Auxiliary experiments indicate that droplet sizes remain constant during oxidation experiments (up to 16 days) [25].

### 3.2. Determination of the Partition Constants of HT and Its Derivatives in Intact Fish Oil Emulsions and Nanoemulsions

The values of the partition constants *P*_O_^I^ and *P*_W_^I^ listed in Table 1 were determined in intact HEn emulsions and nanoemulsions by fitting the variations of observed rate constant *k*_obs_ with Φ_I_. Details on how these calculations are carried out can be found elsewhere [12,13,14,15,26,27] and some representative plots can be found in Figure S3, Supplementary Data. In all cases, *P*_O_^I^ and *P*_W_^I^ values are >1, which means that HT and its derivatives have a natural affinity for the fish oil–water interface since the Gibbs free energy is negative. However, the tendency to be incorporated into the interfacial region is different for each AO because the *P*_O_^I^ and *P*_W_^I^ values are different.

In both emulsions and nanoemulsions, *P*_W_^I^ values increase with increasing length of the alkyl chain of the HT derivatives, keeping with the expected hydrophobic effect. On the other hand, *P*_O_^I^ values increase upon increasing the hydrophobicity of the HT derivatives, reaching a maximum value for the octyl derivative (HT8) and then decrease as a consequence of the oil being a better solvent than the interfacial region for the most hydrophobic AOs. The results are in line with those previously obtained from other series of antioxidants such as gallic acid, where a parabolic-like variation in *P*_O_^I^ with the length of the alkyl chain was observed [12,13,31].

Interestingly, no significant differences were detected in the *P*_O_^I^ and *P*_W_^I^ values obtained either in emulsions and nanoemulsions, Table 1, with differences lower than 15%, suggesting that AO distributions are not affected by changes in droplet size.

Moreover, the values of the intrinsic rate constant *k*_I_ (for the reaction between the probe and the AOs in the interfacial region) are independent of the hydrophobicity of hydroxytyrosol derivarives and independent of the droplet size. The *k*_I_ = 21.9 ± 2.6 M^−1^s^−1^ value obtained in nanoemulsions must be considered basically the same as that obtained in emulsions (*k*_I_ = 17.3 ± 1.4 M^−1^s^−1^) because their differences are lower than 25%, in systems where the droplet size changed ~5–10 fold. This similarity in the *k*_I_ values suggests that the phenolic moieties of the AOs are located in environments with similar solvent properties in both coarse emulsions and nanoemulsions [15,25] and that the medium properties of the interfacial regions of emulsions and nanoemulsions are basically the same [32].

### 3.3. Distribution of HT and Its Derivatives in Fish Oil-in-Water Emulsified Systems

**Figure 2:**
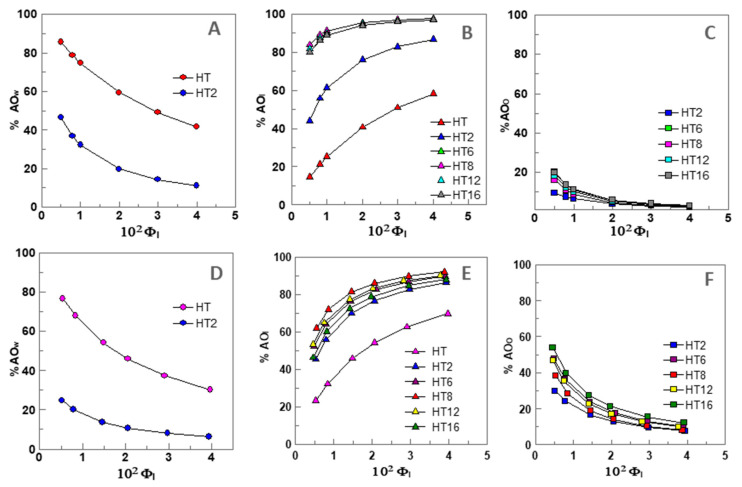
Variation of the percentages (%AO) of HT and its derivatives in the different regions (W—water, I—Interface, O—oil) of 1:9 (O/W) nanoemulsions ((**A**–**C**), [AO_T_] = 0.125 mM) and 4:6 (O/W) emulsions ((**D**–**F**), [AO_T_] = 0.500 mM) composed of fish oil/0.04 M citrate buffer, pH = 3.65/Tween 80, T = 25 °C.

Figure 2A–C shows the distribution of AOs in different regions of 1:9 O/W nanoemulsions as a function of the surfactant volume fraction Φ_I_.

Figure 2A shows that only HT (~85%) and HT2 (~47%) are present in the aqueous region at Φ_I_ = 0.005. The high value obtained for HT is in keeping with its high solubility in water [28]. The percentage of both HT and HT2 decrease upon increasing Φ_I_ to %HT_W_ ~43% and %HT2_W_ ~10% when Φ_I_ = 0.04. All AOs, except HT, are present in the oil region, Figure 2C, but their percentages (%AO_O_) at Φ_I_ = 0.005 are less than ~20%, and %AO_O_ decreases upon increasing Φ_I_. Figure 2B shows that all AOs are present in the interfacial region and their percentage increases upon increasing Φ_I_. Only ~15% of HT is present at Φ_I_ = 0.005, however, the percentage of the most hydrophobic derivatives (HT8, HT12, and HT16) is higher than 80%.

What is important to emphasis, is that the results in Figure 2A–C highlight the strong effect that the emulsifier volume fraction has on the percentage of AOs in the various regions of the emulsions. Especially significant are the variations observed in the percentage of AOs in the interfacial region: all AOs are spontaneously incorporated in this region so that the higher the Φ_I_ value corresponds to the higher percentage of AO in the interfacial region. Similar trends were obtained for 4:6 O/W emulsions as expected from the similar distribution constants obtained for all compounds in both systems (Figure 2D,F).

### 3.4. Aqueous, Interfacial, and Oil Concentrations of HT and Its Derivatives in Fish Oil Emulsified Systems

**Figure 3:**
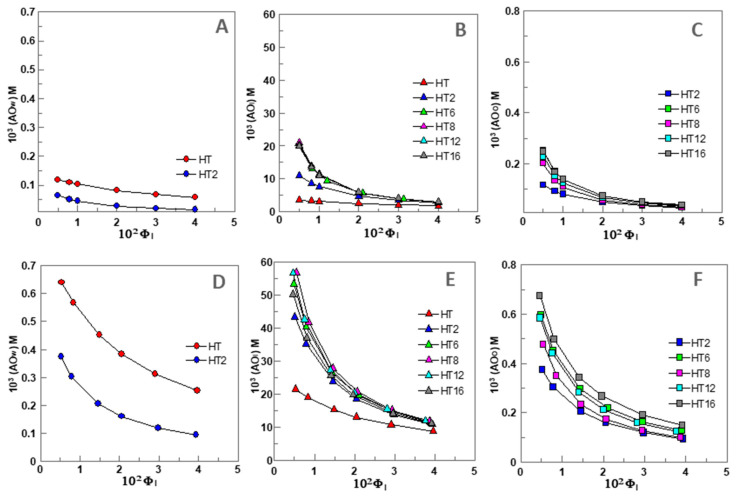
Variation of the concentration of HT and its derivatives (AO) in the different regions (W—water, I—Interface, O—oil) of 1:9 (O/W) nanoemulsions ((**A**–**C**), respectively, [AO_T_] = 0.125 mM) and 4:6 (O/W) emulsions ((**D**–**F**), respectively, [AO_T_] = 0.500 mM) composed of fish oil/0.04 M citrate buffer, pH = 3.65/Tween 80, T = 25 °C.

Because AOs partition between the aqueous, interfacial, and oil regions in different extents, their “effective” concentrations in each region should be distinct from the stoichiometric concentration [AO_T_], since the volumes of each region are not the same. Thus, we determined the effective concentrations of HT and its derivatives in the different regions of 1:9 O/W fish oil-in-water emulsions and nanoemulsions (expressed as moles of AO per liter of the particular region) as indicated in Section 2.6. Their variations for 1:9 (O/W) nanoemulsions with Φ_I_ are illustrated in Figure 3A–C. Similar variations were found for 4:6 O/W emulsions (Figure 3D,F).

We note that these concentrations represent the “effective” concentrations in each region which need to be considered when analyzing the rates of the inhibition reaction by antioxidants since the rate of the reactions depend, among others, on the effective concentration of the reactants at the reaction site [12,13,14,15,25,26,31].

Preliminary analysis of the results in Figure 3A–C show that the effective antioxidant concentration may be higher or lower than the stoichiometric concentration ([AO_T_] = 0.125 mM) because of the differential partitioning of the antioxidants and the different volumes of each region. For example, the effective concentrations of HT and HT2 in the aqueous region, Figure 3A, are slightly lower or 2-fold lower (Φ_I_ = 0.005) than the stoichiometric concentrations and decrease upon increasing Φ_I_, so that the effective concentrations at Φ_I_ = 0.04 are ~2 (HT) and ~10 times (HT2) lower than [AO_T_].

The effective concentration in the oil region, Figure 3C, may be higher (up to 2-fold) or much lower than [AO_T_], depending on the liposolubilty of the AOs, but higher than that in the aqueous region. For instance, at Φ_I_ = 0.005, the effective concentrations of the most hydrophobic AO (HT16) is 2 times higher than [AO_T_] but that of HT2 is equal. However, for any of the AOs, the effective concentration in the oil region decreases upon increasing Φ_I_, and at Φ_I_ = 0.04 becoming 3–7 times lower than [AO_T_].

The effective interfacial concentration is, in contrast, much higher than the stoichiometric concentration (20–170 times). However, it also decreases up to 8 times upon increasing Φ_I_, Figure 3B. Therefore, results indicate that AOs are concentrated in the interfacial region and the lower the surfactant concentration employed in the preparation of the emulsion, the higher the effective interfacial concentration.

It is worth noting that this variation contrasts with that obtained for the variation of %AO_I_, Figure 2B. This apparent contradiction can be rationalized on the basis of Equation (6), which predicts that, for any given [AO_T_], the effective concentration of the AO in the interfacial region depends on both the percentage of AO in this region and the emulsifier volume fraction Φ_I_. An increase in Φ_I_ increases the fraction of AOs in the interfacial region but the interfacial volume also increases. For example, %HT8_I_ increases from ~84% to 98% (~1.2 fold) on going from Φ_I_ = 0.005 to Φ_I_ = 0.04 but (HT8_I_) decreases by ~8 fold.

### 3.5. Effects of the Emulsifier Volume Fraction, and Oil to Water Ratio on the Effective Interfacial Concentrations of HT and Its Derivatives in Emulsions and Nanoemulsions

In attempting to improve the antioxidant efficiency, we analyzed the effects of the surfactant volume fraction and the oil to water ratio employed in the preparation of the emulsion. These parameters are important because they may modulate the interfacial concentrations of the AOs as we demonstrated in previous works, where we found that there is a positive correlation between the interfacial antioxidant concentrations and their antioxidant efficiency [12,14,15,27].

**Figure 4:**
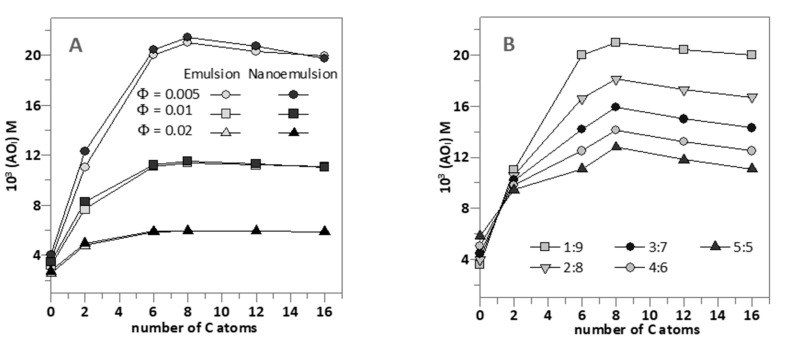
(**A**) Comparative interfacial concentrations of hydroxytyrosol and its derivatives (AO_I_) in 1:9 (O/W) emulsions (● − Φ_I_ = 0.005, ■ − Φ_I_ = 0.01, ▲ − Φ_I_ = 0.02) and 1:9 (O/W) nanoemulsions (● − Φ_I_ = 0.005, ■ − Φ_I_ = 0.01, ▲ − Φ_I_ = 0.02) at different emulsifier volume fractions (Note the overlapping dark grey and light grey symbols). (**B**) Effect of O/W ratio on the interfacial antioxidant concentrations (AO_I_) in nanoemulsions at Φ_I_ = 0.005. In both figures, [AO_T_] = 0.125 mM.

Figure 4A shows the effects of the surfactant volume fraction Φ_I_ on the interfacial concentrations (expressed as moles of AO per liter of interfacial region) of the AOs studied in emulsions and nanoemulsions.

No significant differences in the interfacial concentrations of the antioxidants were detected between emulsions and nanoemulsions at any given Φ_I_, demonstrating the negligible effect of the droplet size on the effective concentrations of antioxidants. As indicated in the section above, for any of the AOs investigated, the interfacial concentration decreases with increasing Φ_I_ because of the dilution effect exerted by the increase in the interfacial volume.

It is worth noting the effects of increasing the hydrophobicity of the AOs on the interfacial concentrations. (AO_I_) increases upon increasing the alkyl chain length of the AO up to a maximum (HT8) after which a further increase in the hydrophobicity results in a decrease of the effective interfacial concentration. This parabolic-like effect, which is much more notorious at low Φ_I_ (e.g., 0.005, Figure 4A) than at high Φ_I_ values, is usually known as the cut-off effect and, as we previously demonstrated, it is a consequence of the increased solubility of the most hydrophobic AOs in the oil with respect to that in the interfacial region [12,14,15,26,27,33]. These results are in line with those obtained for the interfacial area in 1:9 O/W emulsions using a low energy methodology as the HT8 showed to have the highest influence in the formation of the oil droplet interface.

Figure 4B shows the strong influence of the O/W ratio employed in the preparation of nanoemulsions on the interfacial concentrations of the AOs at constant Φ_I_ = 0.005 (the lowest amount employed). Two different behaviors are observed: the interfacial concentration of HT (and probably that of H1) increases slightly upon increasing the O/W ratio. Meanwhile, for the more hydrophobic AOs, the interfacial antioxidant concentrations decrease ~2 fold upon increasing the O/W ratio from 1:9 to 5:5. We note that the interfacial AO concentrations are directly related to the efficiency of AOs. Hence, results in Figure 4B predict that, for HT derivatives with chain lengths >6 carbon atoms, the higher the oil content is, the lower the interfacial concentration and, thus, the lower the antioxidant efficiency should be expected.

### 3.6. Antioxidant Efficiency of HT and Its Derivatives in Fish Oil-in-Water Emulsions and Nanoemulsions: Key Insights

Figure 4A,B show the effects of Φ_I_ and of the O/W ratio on the interfacial concentrations of the AOs. Changes in (AO_I_) should modify the efficiency of the AOs since the rate of the inhibition of the lipid oxidation reaction with antioxidants (LOO^●^ + AO → LOOH + AO^●^) depends on the effective concentration of AOs at the reaction site, which is the interfacial region, and on the rate constant *k*_inh_ for the reaction of lipid radicals with the AOs. This should be the same for all AOs because (i) they have the same reactive moiety that is located in a similar environment (see Section 3.2) and (ii) the length of the grafted alkyl chain does not modify *k*_inh_. We previously demonstrated this hypothesis [12,15,26,27] and further corroborated it here. Table 1 shows that all compounds have similar EC_50_ values, indicating a negligible effect of the length of the alkyl chain of HT derivatives on their reactivity with DPPH^●^. These results are confirmed by cyclic voltammetry, since no significant difference (*p* < 0.05) in the values for the first anodic potential of all HT esters was detected either in the presence or absence of emulsifier (Table 1).

**Figure 5:**
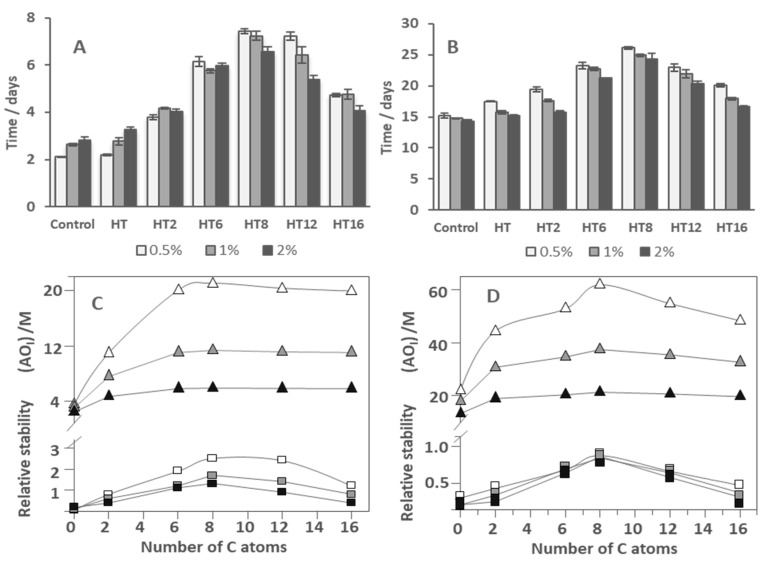
Effects of the AO chain length on the oxidative stability of fish oil 1:9 (O/W) nanoemulsions ((**A**), [AO_T_] = 0.125 mM) and 4:6 O/W emulsions ((**B**), [AO_T_] = 0.500 mM) prepared with Φ_I_ = 0.005, 0.01, and 0.02 in terms of the time required to reach an increase of 0.5% ΔCD with time. Comparison between the antioxidant efficiency of AOs in 1:9 O/W nanoemulsions (**C**) and 4:6 O/W emulsions (**D**) and the values of (AO)_I_ in the same systems, at Φ_I_ = 0.005, 0.01, and 0.02.

As in previous works [12,14,31], the efficiency of AOs in 1:9 O/W nanoemulsions and 4:6 O/W emulsions at T = 40 °C was evaluated (Figure S4, Supplementary Data). Figure 5A,B shows the time (days) needed to reach a percentage of conjugated dienes of 0.5% in both systems. A parabolic-like variation in the AO efficiency with the alkyl chain length is observed, which is similar to that previously obtained in olive oil emulsions [28].

As shown here in Figure 5C,D and elsewhere, the variation of the oxidative stability with the length of the alkyl chain parallels that obtained for the variation of the (AO_I_) [28]. Figure 5A–D also shows the effect in both systems of the surfactant volume fraction Φ_I_. The general picture obtained is that, in both systems, for any of the AOs with a chain length >2 carbon atoms, an increase in Φ_I_ results in a decrease in the oxidative stability in line with the observed decrease in the (AO_I_), Figure 5A,B. However, results also show that there is no correlation between particle size and nanoemulsion oxidative stability. On one hand, in the absence of AO, the 4:6 O/W emulsions showed the same oxidative stability regardless of the droplet size (Figure 5B). On the other hand, at first sight, results for nanoemulsions (Figure 5A) are contradictory and depend on the ingredients and their concentration. In 4:6 O/W emulsions and in 1:9 O/W nanoemulsions in the presence of AOs more concentrated at the interface region (HT8, HT12, and HT 16), a decrease in the droplet diameter seems to decrease the oxidative stability of the system. This can be related to the higher total surface area of these smaller droplets (Figure 5A,B). However, and in contrast, in the case of control and HT loaded nanoemulsions (Figure 5A), we observed an increase in the stability even with increasing surface area. These results were also found in our previous work in 1:9 O/W nanoemulsions loaded with gallic acid and its more hydrophilic derivatives [15], corroborating the idea that there is no correlation between droplet size and oxidative stability.

We need to have in mind that during oxidation, the rate of any chemical reaction depends on the concentration of the reactants and not on the reaction surface. Only in the case of reactions where a transfer between phases occurs, will the speed of the reactions be affected (reactions in heterogeneous systems). This is not the case in an emulsified system, since each region of this system (interphase, oil and aqueous), besides having different properties, cannot be considered as a true phase as their limits are not clearly defined. Therefore, any factor that changes reactant concentrations at the reaction site (the interfacial region) will affect the oxidative stability of the emulsified system.

With the increase in the emulsifier fraction, we will also have a dilution of radicals at the interface region. This fact would increase the oxidative stability of the emulsified system in the absence of AO which we can actually observe in 1:9 O/W nanoemulsions (Figure 5A) [15]. On the other hand, and as we have previously discussed, in the presence of an AO, not only the radicals will be diluted with the increase in the surfactant fraction, but also the AO will be diluted. These dilutions will decrease both the reaction rate between the radicals and the AO and the reaction rate between radicals and substrates (lipid oxidation) and, therefore, the balance in the effect of these dilutions will determine the final oxidative stability of the emulsified system.

These results point to the fact that several factors must be taken into account when trying to predict whether there will be an increase or a decrease in the oxidative stability of an emulsified system. This can be observed for the stability of 1:9 O/W nanoemulsions loaded with HT, HT2, and HT6 (Figure 5A) that show a different behavior with the increase in the emulsifier fraction when compared with 4:6 O/W emulsions loaded with the same compounds (Figure 5B). Because HT is mostly distributed in the aqueous region in 1:9 W/O nanoemulsions at 0.05 emulsifier fraction and its concentration at the interface region is very low (Figure 3B), HT does not show any antioxidant activity in this system. In this case, the dilution of the HT with the increasing emulsifier fraction will not have a consequence in the nanoemulsion stability. However, this increase in Φ_I_ will decrease the radicals’ concentration, justifying the greater stability of HT loaded nanoemulsions prepared with an emulsifier fraction of 0.01 and 0.02 (Figure 5A). Nevertheless, this hypothesis will need further investigation in the future.

In 4:6 O/W emulsions at Φ_I_ = 0.005 loaded with HT, the concentration of HT at the interfacial region is much higher than in the nanoemulsion (Figure 3E) since the higher O/W shifts the HT from the water region (Figure 3D) to the interface region. In these emulsions, since the interfacial concentration of HT is higher than in 1:9 O/W nanoemulsions, we were able to observe some antioxidant activity (*p* < 0.05) (Figure 5B). However, when the emulsifier fraction is increased, the deep decrease in the HT interfacial concentration results in the absence of antioxidant activity for higher emulsifier fractions. These results agree with those previously obtained for gallic acid and its esters in fish oil-in-water nanoemulsions [15] and with literature reports [12,14,15,27,32]. Thus, regardless of the droplet size, there is a direct relationship between (AO_I_) and its antioxidant efficiency (Figure 5C,D).

In the past, changes in the oxidative stability of emulsions with different droplet sizes were attributed to the differential surface areas of the droplets in the studied system. However, contradictory reports on the precise effects of the droplet size on the antioxidant efficiency were reported [18,21]. Our results corroborate the idea that the droplet size has a negligible effect on the oxidative stability of emulsified systems. The main determinant of the oxidative stability of the emulsions is the balance of factors that changes the effective concentration of reactants, AOs, and radicals, at the reaction site (which is the interfacial region) [12,14,15,25,26,27,31].

## 4. Conclusions

**Figure 6:**
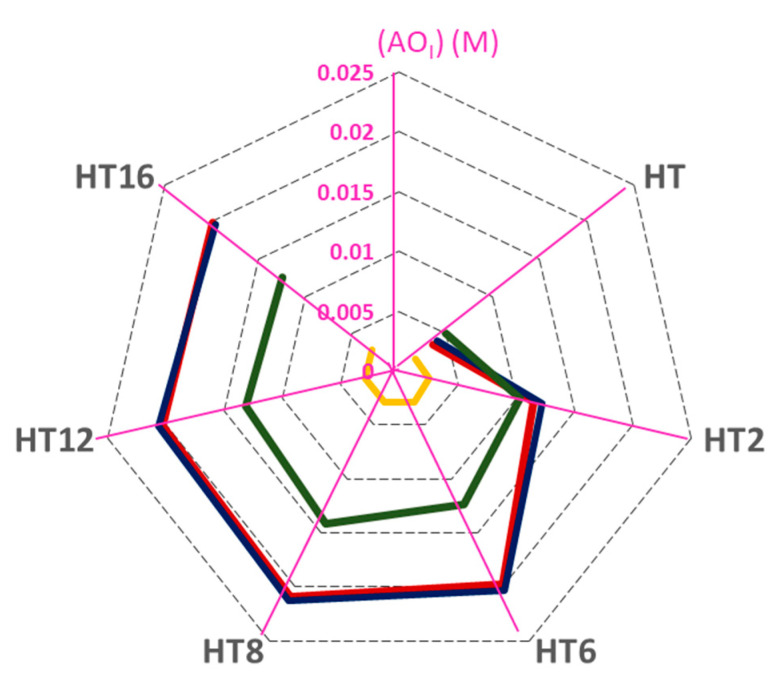
Radar chart presentation for the analysis of the effect of droplet size, O/W ratio, and emulsifier concentration on the interfacial antioxidant concentrations (from 0 to 0.025 M) which are indicated in each axis purple lines, expressed in terms of molarity (1:9 (O/W) emulsion at Φ_I_ = 0.005; 1:9 (O/W) nanoemulsion at Φ_I_ = 0.005; 4:6 (O/W) nanoemulsion at Φ_I_ = 0.005; 1:9 (O/W) nanoemulsion at Φ_I_ = 0.02). [AO_T_] = 0.125 mM in 1:9 (O/W) emulsified systems and [AO_T_] = 0.500 mM in 4:6 (O/W) emulsified system.

To summarize the results obtained and to get a better understanding of how the different parameters analyzed affect the interfacial molarity of AOs and their efficiency, we mapped the variations of the tested parameters in a radar-like plot, Figure 6.

The radar chart is aimed at providing a rapid visualization of how interfacial concentrations change upon changing both the experimental conditions and the hydrophobicity of the antioxidants. For this purpose, we plot concentric heptagons, each one standing for a given interfacial molarity (which is indicated in the scale). The upper corner of the heptagon represents the interfacial concentration and each of the other corners represents a particular antioxidant. For the sake of clarity, radial straight lines emerging from the center of the heptagons are drawn and the corners are connected to each other (grey dashed lines in the chart). The solid lines in the chart represent different experimental conditions (given in the figure caption) so that one can easily envisage: (1) the changes in the interfacial concentrations for each antioxidant (i.e., the hydrophobicity effects) under the same experimental conditions, and (2) rapidly assess, for a given antioxidant, the effects of experimental conditions on the values of its interfacial molarity. As observed in Figure 6, the droplet size has no effect on (AO_I_) as the red and blue lines in Figure 6 overlap. For all derivatives, the emulsifier volume fraction shows the largest impact on the interfacial antioxidant concentration. For example, the red and yellow lines show that, for HT8, (AO_I_) decreases ~7 fold on going from Φ_I_ from 0.005 to 0.02. The O/W ratio has a smaller effect than emulsifier volume fraction on the interfacial AO concentrations for hydrophobic derivatives and an apparent slight impact on the interfacial AO concentrations for hydrophilic derivatives. For example, for HT8, (AO_I_) decreases ~1.5-fold with increasing O/W from 1:9 to 4:6 (red and green lines in Figure 6).

Results show that droplet size has no effect on the AO interfacial concentrations and that the main parameter controlling the (AO_I_) is the emulsifier volume fraction, having the O/W ratio a smaller effect (Figure 6). Indeed, partitioning depends on the intermolecular forces, the hydrogen-bonding ability of the AOs and solvating properties of the different regions of the emulsion. Thus, more work is necessary since, unfortunately, we still cannot predict quantitatively how antioxidants are distributed between the different regions of emulsified systems. However, with the aid of the pseudophase kinetic model, we can quantitatively determine the interfacial concentrations of the antioxidants and correlate them with the antioxidant efficiencies, providing a robust and trustable method to grasp the complex behavior of antioxidants in emulsions containing different droplet sizes. This opens new possibilities and strategies to control the lipid oxidation problem and to avoid the oxidative spoilage of foods and pharmaceutical formulations.

## Figures and Tables

**Table 1:** AO percentages in aqueous phase (%AO_W_) and *P*_W_^O^ values in binary fish oil-water systems. *P*_O_^I^ and *P*_W_^I^ values and the rate constant in the interfacial region, *k*_I_, in fish oil emulsified systems. EC_50_ (mole AO/mole DPPH^●^) and anodic potentials *E_pa_* versus Ag–AgCl measured at a glassy carbon electrode for 10^−4^ mol L^−1^ solutions of compounds in buffer solution in the absence and presence of 2% Tween 80. Emulsion droplet sizes of emulsified systems containing AOs in nm.

		HT	HT2	HT6	HT8	HT12	HT16
**Binary** **System**	(%AO_W_)	99.45 ± 0.5	83.3 ± 1.1	≈0	≈0	≈0	≈0
	*P*_W_^O^	0.008 ± 0.001	1.80 ± 0.01	---	---	---	---
**Emulsion** **1:9 O/W**	*P*_W_^I^	34 ± 1	207± 39	---	---	---	---
	*P*_O_^I^	---	115 ± 22	89 ± 8	119 ± 45	97 ± 14	75 ± 12
	10^2^k_I_(M^−1^s^−1^)	10.6 ± 0.2	16.7 ± 5.7	19.0 ± 0.3	18.7 ± 8.8	16.3 ± 0.4	16.0 ± 0.2
**Nanoemulsion** **1:9 O/W**	*P*_W_^I^	30 ± 4	186 ± 14	----	----	----	----
	*P*_O_^I^	----	103 ± 8	79 ± 22	104 ± 13	89 ± 19	80 ± 13
	10^2^k_I_(M^−1^s^−1^)	19.0 ± 1.3	20.7 ± 2.9	25.6 ± 0.6	23.6 ± 0.2	20.2 ± 0.4	19.6 ± 0.3
**EC_50_^I^**	5 min	0.323 ± 0.005	0.354 ± 0.009	0.300 ± 0.003	0.295 ± 0.005	0.345 ± 0.005	0.331 ± 0.011
	15 min	0.287 ± 0.004	0.309 ± 0.007	0.272 ± 0.003	0.265 ± 0.005	0.319 ± 0.004	0.305 ± 0.006
	60 min	0.258 ± 0.004	0.281 ± 0.004	0.247 ± 0.003	0.239 ± 0.003	0.294 ± 0.006	0.298 ± 0.007
**E_pa_ (V)**	0% Tween 80	0.418	0.421	0.404	0.412	0.402	0.403
	2% Tween 80	----	----	0.388	0.419	0.382	0.394
**Emulsion droplet size** **(Φ_I_ = 0.5, nm)**	1:9 O/W (LEn) ^II^	8402 ± 162 ^a^	8564 ± 197 ^a^	6634 ± 128 ^b^	5046 ± 114 ^c^	5689 ± 134 ^d^	7824 ± 179 ^e^
	1:9 O/W (HEn) ^III^	1280 ± 60	1227 ± 57	1168 ± 61	1145 ± 45	1189± 37	1235 ± 78
	4:6 O/W	4610 ± 203	4551 ± 176	4465 ± 155	4330 ± 117	4501 ± 187	4534 ± 144

^I^ Data extracted from Almeida et al., 2016 [27]. ^II^ Emulsions obtained with the aid of a vortex (LEn, low energy method). ^III^ Emulsions obtained by high-speed homogenization (HEn, high energy method). Different letters within a row indicate samples that were significantly different (*p* < 0.05).

**Table 2:** Theoretical calculations (assuming that all droplets have the same size within each emulsified system) of some physical characteristics of the prepared (O/W) fish nanoemulsions in the absence of compounds.

	Nanoemulsions			Emulsions		
**Φ_O_**	1.0	1.0	1.0	4.0	4.0	4.0
**10^2^ Φ_I_**	0.5	1.0	2.0	0.5	1.0	2.0
**ζ-potential (mV)**	−18.1	−14.2	−13.5	−22.6	nd *	nd *
**10^6^ d (m)**	0.304	0.231	0.166	4.71	3.12	2.73
**10^12^ S_droplet_ (m^2^)**	0.29	0.17	0.09	69.7	30.6	23.4
**10^20^ V_droplet_ (m^3^)**	1.47	0.65	0.24	5468	1589	1065
**10^−12^ N_d_**	68	155	418	0.73	2.52	3.76
**S_total_ (m^2^)**	19.7	26.0	36.1	5.1	7.7	8.8
**10^2^ m_T80 available /_ m^2^ of S (g)**	0.25	0.39	0.55	9.81	1.30	2.28
**10^2^ m_T80, droplet_ (g)**	0.52	0.68	0.95	0.13	0.20	0.23
**10^2^ m_T80, excess_ (g)**	−0.02	0.32	1.05	0.37	0.80	1.80

Φ_O_ = oil volume per 100 g of emulsion; Φ_I_ = surfactant volume fraction; d = droplet diameter; V_droplet_ = volume of one droplet; S_droplet_ = droplet surface; N_d_ = total number of droplets; S_total_ = surface of all droplets; m_T80_, droplet = mol of surfactant required for saturation per 100g of emulsion (calculated by employing an interfacial coverage at saturation of Γ∞ = 2 × 10^−6^ (mol m^−2^); m_T80_,_excess_ = excess mol of Tween 80 remaining in the aqueous phase; m_T80 available_, mol of Tween 80 used. * nd—no determined.

## Supplementary Materials

The following are available online at https://www.mdpi.com/2304-8158/9/12/1897/s1, Figure S1: Illustrative variations of absorbance of azo dyes from the reaction between chemical probe and NED with time. Figure S2: Illustrative variations of the observed rate constant for the reaction between some HT derivatives and chemical probe with the emulsifier volume fraction. Figure S3: Interfacial areas (IA) expressed as m^2^/mL of emulsion of 1:9 (O/W) emulsions obtained by a low energy method loaded with antioxidants prepared with Φ_I_ of 0.005. Figure S4: Effects of the AO chain length at selected Φ_I_ on the lipid oxidation reaction kinetic in emulsified systems, Scheme S1: Values of half-lives, t_1/2_, for the reactions of 16-ArN2^+^ with AOs and *N*-(1-naphthyl)ethylenediamine (NED) obtained under the experimental conditions.
