# Supplementary material for: Interfacial Concentrations of Hydroxytyrosol Derivatives in Fish Oil-in-Water Emulsions and Nanoemulsions and Its Influence on Their Lipid Oxidation: Droplet Size Effects
Source: Foods. 2020 Dec 18;9(12):1897. doi: 10.3390/foods9121897 (PMC7765823; doi:10.3390/foods9121897)
Supplement: Supplementary file 1 [file foods-09-01897-s001.pdf]

Interfacial Concentrations of Hydroxytyrosol Derivatives in Fish Oil-in-Water Emulsions and Nanoemulsions and Its Influence on Their Lipid Oxidation: Droplet Size Effects

Marlene Costa [1], Sonia Losada-Barreiro [1,2], Carlos Bravo-Díaz [2], Luís S. Monteiro [3] and Fátima Paiva-Martins [1,*]

1. Determination of interfacial areas in emulsions loaded with HT derivatives by a turbidimetric method

Interfacial areas (IA) of 1:9 (O/W) emulsions obtained by a low energy method loaded with antioxidants prepared with φ_I of 0.005 were evaluated by employing a turbidimetric method as described by Pearce and Kinsella [3]. The interfacial area (IA) of the dispersed oil phase were calculated according to Cameron [4] and expressed as m^2/mL of emulsion. The calculated interfacial areas (IA) were then use to calculate the average droplet size in these emulsions.

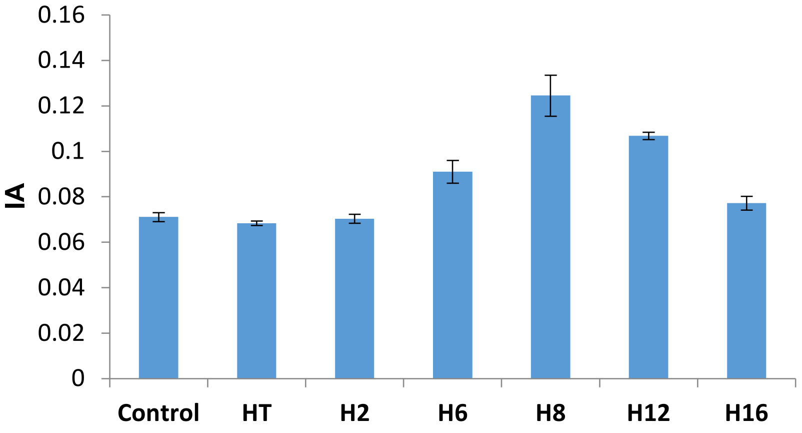

Figure S1. Interfacial areas (IA) expressed as m^2/mL of emulsion of 1:9 (O/W) emulsions obtained by a low energy method loaded with antioxidants prepared with φ_I of 0.005.

2. Determination of the observed rate constant, k_{obs}, for the reaction between 16-ArN_2^+ and the AOs in fish oil emulsified systems.

The reaction between 16-ArN_2^+ and AOs in each region of emulsified systems is the product of the second-order rate constant and the concentration of each reactant in that region in moles per liter of region volume. 16-ArN_2^+ Has a long hydrophobic alkyl chain and a cationic headgroup and is both water and oil

insoluble. Its reactive $-N_2^+$ group is located in the interfacial region, where it reacts with AOs, as illustrated in Figure 1, and its concentration in the oil and water regions can be considered negligible. Under pseudo-first order conditions (being [AO] much higher than [16-ArN$_2^+$]), the observed rate is given by Equation (S1).

$$rate = k_{obs}\left[16-ArN_{2\,T}^{+}\right] = k_2\left[16-ArN_{2\,T}^{+}\right]\left[AO\right] = k_I\left(16-ArN_{2\,I}^{+}\right)\left(AO_I\right)\Phi_I \quad \text{(S1)}$$

In Equation (S1), k_2 and k_I are the observed second rate constant and the second order rate constant in the interfacial region, respectively; k_{obs} is the observed overall rate; square brackets, [], denote the concentration in mol/L of the total emulsion volume; the subscript T stands for total; parentheses, (), denote the concentration in mol/L of the volume of a particular region; subscript I stands for the interfacial region; and Φ_I is the emulsifier volume fraction defined as the ratio of the volume of emulsifier divided by the total volume of the emulsion ($\Phi_I = V_{surf}/V_{Total}$).

The reaction between 16-ArN$_2^+$ and derivatives of hydroxytyrosol, Scheme S1, was followed spectrometrically by employing the dye derivatization method (azo dye formation) described in detail elsewhere [2]. The methodology exploits the rapid reaction of 16-ArN$_2^+$ ions with a suitable coupling agent such as *N*-(1-naphthyl)ethylenediamine dihydrochloride, NED, yielding a stable azo dye whose absorbance can be determined spectrometrically at λ = 572 nm, after dilution with an alcoholic mixture. Solutions of the coupling reagent (NED) were prepared in a 50:50 (v/v) BuOH:EtOH mixture to give [NED] = 0.02 M.

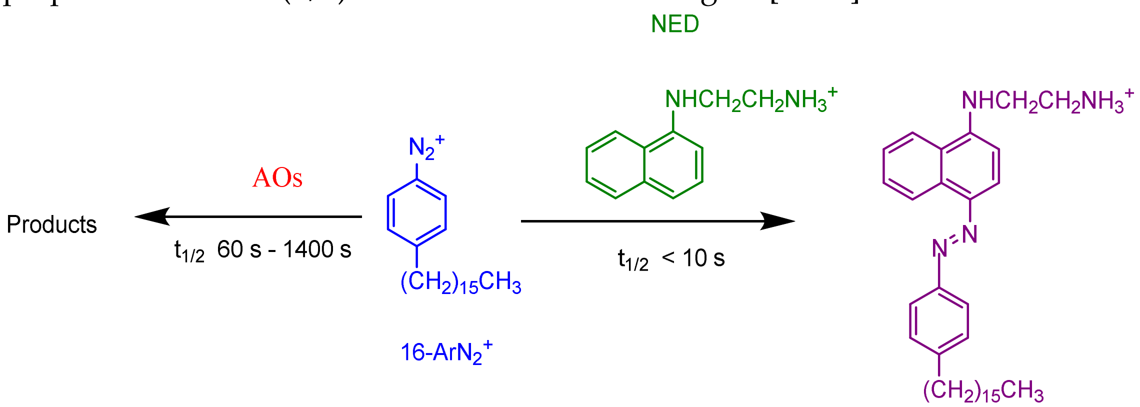

Scheme S1. Values of half-lives, $t_{1/2}$, for the reactions of 16-ArN$_2^+$ with AOs and *N*-(1-naphthyl)ethylenediamine (NED) obtained under the experimental conditions. Notice that the reaction of 16-ArN$_2^+$ with NED is much faster than with AOs, a requirement to get reliable rate constants by using the derivatization method.

k_{obs} Values were obtained by fitting the absorbance-time pairs of data to the integrated first order Eq. S2.In Eq. S2, A_t, A_o and A_{inf} are the measured absorbance at any time, at t = 0 and at infinite time, respectively. An illustrative kinetic plot for the reaction between 16-ArN$_2^+$ and hydroxytyrosol in fish oil-in-water nanoemulsions is shown in Figure S2. Similar kinetic plots were obtained using fish oil-in-water emulsions.

$$\ln\left(A_t - A_{\inf}\right) = \ln(A_0 - A_{\inf}) - k_{obs}t \quad \text{(S2)}$$

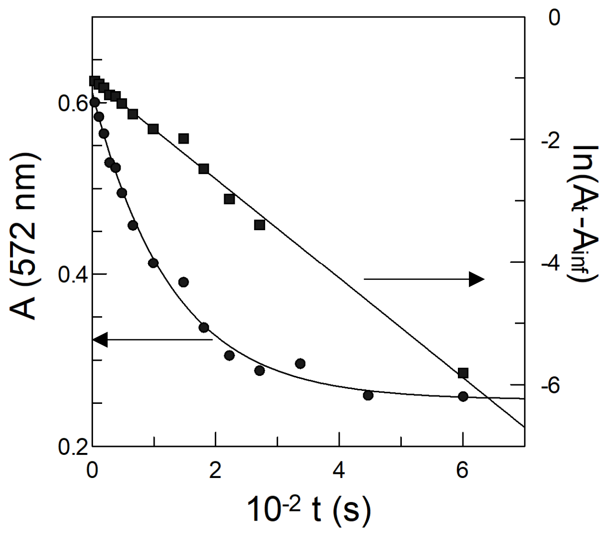

Figure S2. Illustrative variations of absorbance of azo dye from the reaction between chemical probe and NED with the time. Experimental conditions: 1:9 (O/W, vol:vol) fish oil nanoemulsion, Φ_I = 0.0079, [HT] = 2 mM, [$16ArN_2^+$] = 0.29 mM, citrate buffer 0.04M, pH 3.65, T = 25ºC.

3. Determining the partition constants for water-insoluble and oil-insoluble AOs in emulsified systems

For oil-insoluble AOs, i.e., AOs that distribute between the aqueous and interfacial region such as HT, only the constant partition P_W^I is required to define their distribution and equation 4 of the text can be simplified to give equation S3. For water-insoluble AOs, that is, AOs that distribute between the oil and interfacial regions such as derivatives of hydroxytyrosol with alkyl chain length ≥ 6, only the P_O^I partition constant is required and equation 4 of the text can be simplified to give equation S3. Equations 4, S3 and S4 predict that the k_{obs} values should decrease asymptotically with the increase of Φ_I . P_W^I and P_O^I and the interfacial rate constant (kK_I) were evaluated from the slopes and intercepts of the linear fits of 1/k_{obs} *vs* Φ_I to the reciprocals of equations S3 and S4.

$$k_{obs} = \frac{k_I[AO]_T P_W^I}{\Phi_I P_W^I + \Phi_W} \quad \text{(S3)} \qquad k_{obs} = \frac{k_I[AO]_T P_O^I}{\Phi_I P_O^I + \Phi_O} \quad \text{(4}$$

Figure S3 shows a typical plots of the k_{obs} *vs* Φ_I in nanoemulsions. In all cases, k_{obs} decreases by a factor of ~3-10 times by increasing the emulsifier fraction from Φ_I = 0.005 to Φ_I = 0.04 consistent with equations 4, S3 and S4. The solid lines are the

theoretical curves obtained by fitting the experimental data to the equations derived from the pseudophase kinetic model. Similar plots of ~~the~~ k_{obs} vs Φ_I were obtained for fish oil-in-water emulsions.

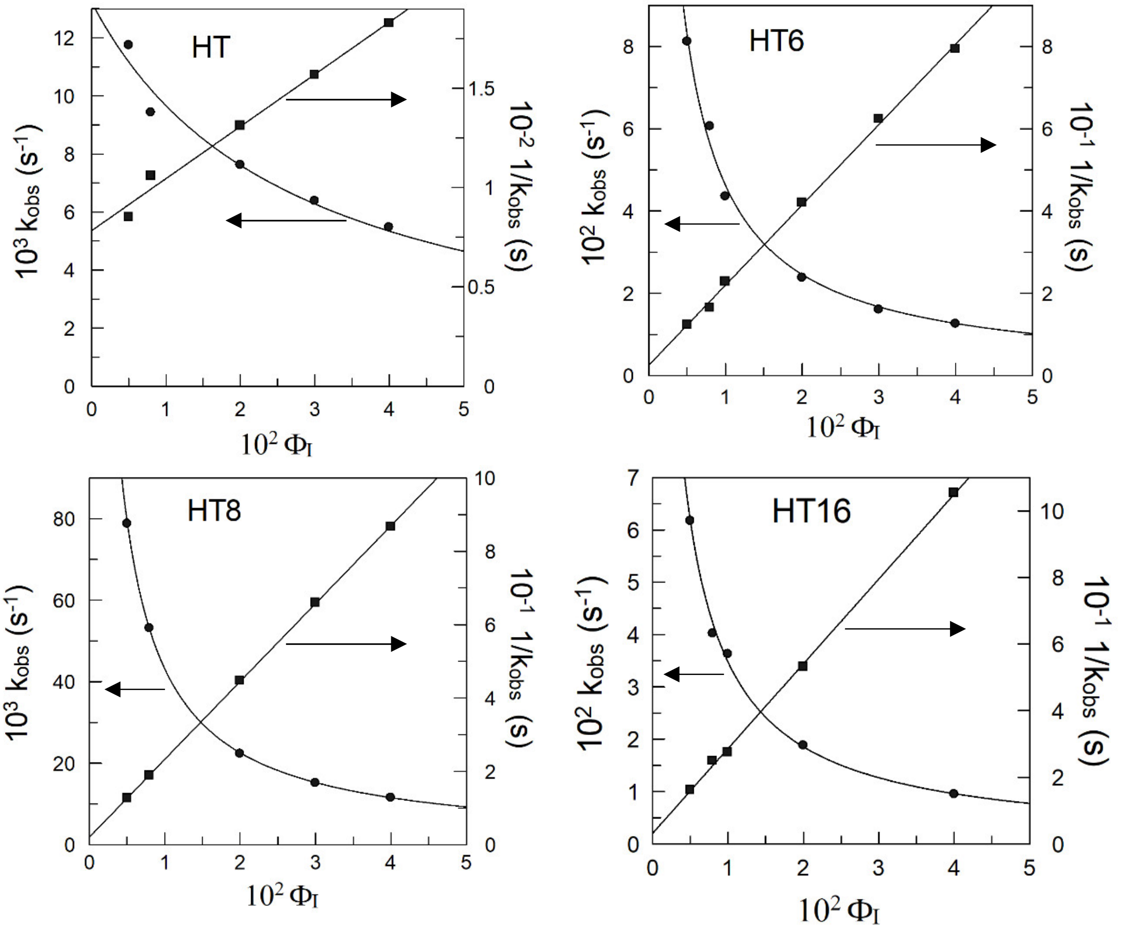

Figure S3. Illustrative variations of the observed rate constant for the reaction between some HT derivatives and the chemical probe with the emulsifier volume fraction. Experimental conditions: 1:9 (O/W, vol:vol) fish oil nanoemulsion, [AO_T] = 2 mM, [$16ArN_2^+$] = 0.29 mM, citrate buffer 0.04M, pH 3.65, T = 25ºC.

4. Effects of the AO chain length at selected ⚜ɪ on the lipid oxidation reaction kinetics in emulsified systems.

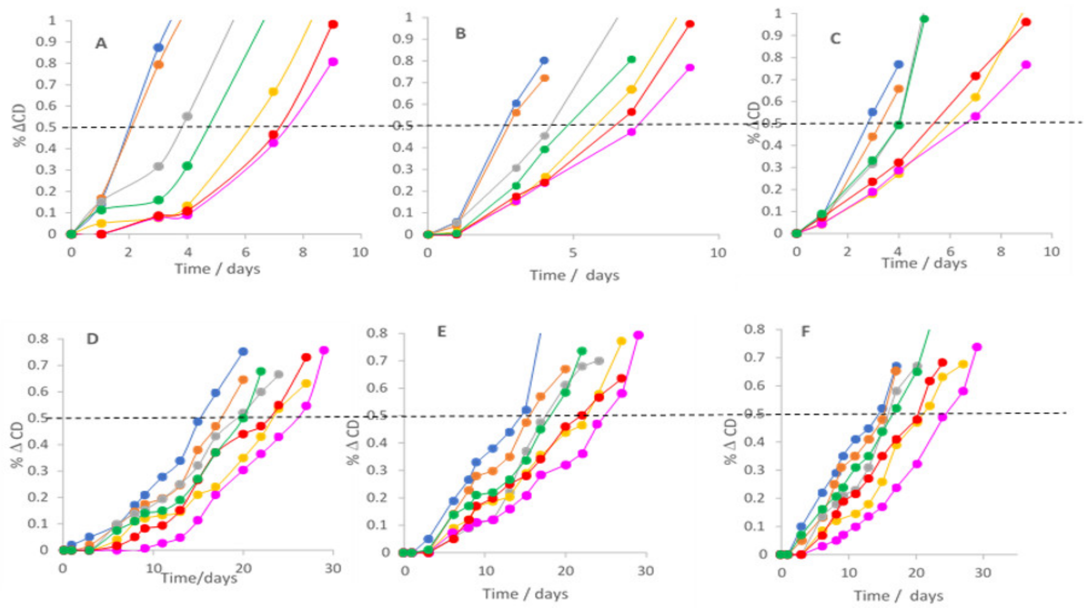

Control-●-; HT -●-; HT2 -●-; HT6-●-; HT8-●-; HT12-●-; HT16-●-

Figure S4- Effects of the AO chain length at selected ◎ɪ on the lipid oxidation reaction kinetics in fish oil 1:9 (O/W) nanoemulsions (A◎◎◎◎ɪ=0.005; B◎◎◎◎ɪ=0.01 and C◎◎◎◎ɪ=0.02; $[AO_T]$= 0.125 mM, T = 40 ºC), and 4:6 O/W emulsions (D◎◎◎◎ɪ=0.005; E ◎◎◎ɪ=0.01 and F◎◎◎◎ɪ=0.02; $[AO_T]$= 0.500 mM, T = 40 ºC) monitored by the formation of conjugated dienes.

5.References

1. Bravo-Díaz, C.; Romsted, L.S.; Liu, C.; Losada-Barreiro, S.; Pastoriza-Gallego, M.J.; Gao, X.; Gu, Q.; Krishnan, G.; Sánchez-Paz, V.; Zhang, Y.; et al. To Model Chemical Reactivity in Heterogeneous Emulsions, Think Homogeneous Microemulsions. *Langmuir* **2015**, *31*, 8961–8979.
2. Sánchez-Paz, V.; Pastoriza-Gallego, M.J.; Losada-Barreiro, S.; Bravo-Díaz, C.; Gunaseelan, K.; Romsted, L.S. Quantitative determination of α-tocopherol distribution in a tributyrin/Brij 30/water model food emulsion. *J. Colloid Interface Sci.* **2008**, *320*, 1–8.
3. Di Mattia, C.D.; Sacchetti, G.; Mastrocola, D.; Pittia, P. Effect of phenolic antioxidants on the dispersion state and chemical stability of olive oil O/W emulsions. *Food Res. Int.* **2009**, *42*, 1163–1170.
4. Cameron, D.R.; Weber, M.E.; Idziak, E.S.; Neufeld, R.J.; Cooper, D.G. Determination of interfacial areas in emulsions using turbidimetric and droplet size data: correction of the formula for emulsifying activity index. *J. Agric. Food Chem.* **1991**, *39*, 655–659.
